# Single-cell genomics of co-sorted *Nanoarchaeota* suggests novel putative host associations and diversification of proteins involved in symbiosis

**DOI:** 10.1186/s40168-018-0539-8

**Published:** 2018-09-17

**Authors:** Jessica K. Jarett, Stephen Nayfach, Mircea Podar, William Inskeep, Natalia N. Ivanova, Jacob Munson-McGee, Frederik Schulz, Mark Young, Zackary J. Jay, Jacob P. Beam, Nikos C. Kyrpides, Rex R. Malmstrom, Ramunas Stepanauskas, Tanja Woyke

**Affiliations:** 1 DOE Joint Genome Institute, Walnut Creek, CA USA; 2 Oak Ridge National Laboratory, Oak Ridge, TN USA; 3 Department of Microbiology, University of Tennessee, Knoxville, TN USA; 4 Department of Land Resources and Environmental Sciences, Montana State University, Bozeman, MT USA; 5 Department of Microbiology and Immunology, Montana State University, Bozeman, MT USA; 6 Department of Plant Sciences and Plant Pathology, Montana State University, Bozeman, MT USA; 7 Bigelow Laboratory for Ocean Sciences, East Boothbay, ME USA

**Keywords:** *Nanoarchaeota*, Single-cell genomics, Symbiosis, Single nucleotide polymorphisms

## Abstract

**Background:**

*Nanoarchaeota* are obligate symbionts of other Archaea first discovered 16 years ago, yet little is known about this largely uncultivated taxon. While *Nanoarchaeota* diversity has been detected in a variety of habitats using 16S rRNA gene surveys, genome sequences have been available for only three *Nanoarchaeota* and their hosts. The host range and adaptation of *Nanoarchaeota* to a wide range of environmental conditions has thus largely remained elusive. Single-cell genomics is an ideal approach to address these questions as *Nanoarchaeota* can be isolated while still attached to putative hosts, enabling the exploration of cell-cell interactions and fine-scale genomic diversity.

**Results:**

From 22 single amplified genomes (SAGs) from three hot springs in Yellowstone National Park, we derived a genome-based phylogeny of the phylum *Nanoarchaeota*, linking it to global 16S rRNA gene diversity. By exploiting sequencing of co-sorted tightly attached cells, we associated *Nanoarchaeota* with 6 novel putative hosts, 2 of which were found in multiple SAGs, and showed that the same host species may associate with multiple species of *Nanoarchaeota*. Comparison of single nucleotide polymorphisms (SNPs) within a population of *Nanoarchaeota* SAGs indicated that *Nanoarchaeota* attached to a single host cell in situ are likely clonal. In addition to an overall pattern of purifying selection, we found significantly higher densities of non-synonymous SNPs in hypothetical cell surface proteins, as compared to other functional categories. Genes implicated in interactions in other obligate microbe-microbe symbioses, including those encoding a cytochrome bd-I ubiquinol oxidase and a FlaJ/TadC homologue possibly involved in type IV pili production, also had relatively high densities of non-synonymous SNPs.

**Conclusions:**

This population genetics study of *Nanoarchaeota* greatly expands the known potential host range of the phylum and hints at what genes may be involved in adaptation to diverse environments or different hosts. We provide the first evidence that *Nanoarchaeota* cells attached to the same host cell are clonal and propose a hypothesis for how clonality may occur despite diverse symbiont populations.

**Electronic supplementary material:**

The online version of this article (10.1186/s40168-018-0539-8) contains supplementary material, which is available to authorized users.

## Background

*Nanoarchaeota* were first reported in 2002 when Huber and colleagues cultured *Nanoarchaeum equitans*, along with its host *Ignicoccus hospitalis*, from a marine hydrothermal vent [1]; this enabled detailed physiological, ultrastructural, and genomic studies of this unique symbiosis [2–9]. *N. equitans* is an obligate symbiont with a reduced genome [3], attaching to and relying on its host for almost all essential cellular components including amino acids, nucleotides, lipids, and co-factors, which appear to be trafficked via a direct cytoplasmic connection between the cells [2, 4, 10]. Later surveys of 16S rRNA sequences revealed *Nanoarchaeota* living in diverse environments, including marine hydrothermal sediments, terrestrial hot springs in Asia, North America, and New Zealand [11–15], and mesophilic hypersaline environments [11]. *Nanoarchaeota* sequences were also retrieved from cool photic regions of the Yellowstone Lake, although the organisms likely originated from submerged thermal springs [15]. These environments span a variety of temperatures from 4 to greater than 100 °C, and pH values from 3.5 to 8.0, suggesting that *Nanoarchaeota* genomes may be similarly diverse. At spatial scales from a few kilometers to tens of meters, hundreds of different *Nanoarchaeota* OTUs have been recovered [15], some with less than 90% identity to other known *Nanoarchaeota* 16S rRNA sequences [12]. However, *N. equitans* is currently the sole sequenced representative from a marine environment [3], and only two other genomes are available (*Nanopusillus acidilobi* and Nst1, “Nanobsidianus stetteri”), both from hot springs in Yellowstone National Park (YNP) [16, 17].

Phylogenetically, *Nanoarchaeota* are grouped basally in the archaeal tree of life [3] with other lineages of ultra-small Archaea termed DPANN (comprising Diapherotrities, Parvarchaea, Aenigmarchaeota, Nanohaloarchaeota, and Nanoarchaeota) [18, 19]. A number of additional phyla have been added to this group more recently (Woesearchaeota, Pacearchaeota, Micrarchaeota). Although the relationship of DPANN to other archaeal phyla remains somewhat uncertain [19, 20], they share many characteristics, including small genomes, limited metabolic capability, and likely a symbiotic lifestyle [21–23]. Currently available genomic data support a symbiotic common ancestor of marine and terrestrial *Nanoarchaeota* lineages and an ancient divergence of these two groups [17], but it is not known if the common ancestor of DPANN or of all *Nanoarchaeota* was symbiotic.

Initial 16S rRNA surveys revealed that many habitats harboring *Nanoarchaeota* do not contain any of the known hosts, suggesting additional species can serve as hosts [11, 13, 14]. Only three host-symbiont pairs are known: *I. hospitalis* and *N. equitans*, Acd1 “Acidicryptum nanophilum” and “Nanobsidianus stetteri” [17], and *Acidilobus* 7A and *Nanopusillus acidilobi* [16]. Co-occurrence and other analyses have suggested additional hosts (e.g., *Vulcanisaeta, Pyrobaculum* [24]), and from the wide range of temperature, pH, and physiochemical parameters described in *Nanoarchaeota* habitats, it is likely that there are multiple hosts. It is unclear when the radiation of terrestrial *Nanoarchaeota* to different environments and hosts occurred and if any of them have switched their hosts. It is also unknown whether the same species of *Nanoarchaeota* can associate with multiple hosts, or vice versa. Attempts to co-culture *N. equitans* and *N. acidilobi* with different hosts have not been successful [5, 16]. Finally, while host cells with multiple *Nanoarchaeota* attached are frequently observed, we do not know whether genomes of these symbionts associated with a single host are clonal or heterogeneous.

A substantial body of work has been amassed with *N. equitans* and *I. hospitalis*, characterizing in detail their genomic, transcriptomic, proteomic, metabolomic, and ultrastructural interactions [2, 3, 5–8]. When in co-culture with *N. equitans*, *I. hospitalis* reduces the diversity of metabolic precursors, channels more of its energy production towards supporting the symbiont, supplies it with specific amino acid precursors, and perhaps re-routes NADH oxidation pathways to enhance ATP synthesis in *N. equitans* [6, 8]. Even a single attached *N. equitans* cell retards the growth of its host in co-culture, and as they proliferate to densities of > 10 attached cells, *N. equitans* prevents host replication altogether [5]. Further, the exponential and stationary growth phases are out of sync in *N. equitans* and *I. hospitalis*, with *N. equitans* continuing to grow as its host enters stationary phase [5]. Less physiological detail is known for terrestrial *Nanoarchaeota*, but several lines of evidence suggest that they may have fewer deleterious effects or could even be beneficial to their hosts under some conditions. Terrestrial *Nanoarchaeota* have slightly larger genomes than *N. equitans* and a larger repertoire of enzymes involved in carbohydrate metabolism. The overall growth of the host *Acidilobus* sp. 7A is not affected by co-culture with *N. acidilobi*, about half of the host cells do not have any attached *N. acidilobi* in stationary phase [5, 16], and growth kinetics are synchronized in *N. acidilobi* and *Acidilobus* sp. 7A [16]. A comparable ectosymbiosis has been observed between *Actinomyces odontolyticus* and a human oral member of *Saccharibacteria* (candidate division TM7), in which the *Saccharibacteria* are obligate symbionts with high host specificity, but the *Actinomyces* host can live independently [25]. Interestingly, while that oral *Saccharibacteria* behaves as a parasite in most culture conditions, they may be able to disguise or protect their hosts from human immune cells, thus acting as mutualists in a different ecological context [25]. Environmental conditions and the presence of competing organisms may be similarly important in understanding the full range of interactions between *Nanoarchaeota* and their hosts.

*Nanoarchaeota* share some similarities with other known obligate microbial symbionts (e.g., insect endosymbionts), including reduced genomes and reliance on a host [26], but have important differences that may lead to different evolutionary pressures and trajectories [27]. As ectosymbionts, *Nanoarchaeota* have access to external sources of DNA including lateral gene transfer with their hosts [7] and with other *Nanoarchaeota* via viral transduction [28, 29]. They have also retained a full suite of genes for DNA recombination and repair [3, 16, 17], and RNA-Seq data from *N. equitans* [30] suggests that genome fragmentation, inversion, re-arrangement, and splitting of protein-coding genes [3, 17, 28] are ongoing processes in *Nanoarchaeota* genomes. Together with large population sizes [28], these factors likely prevent the bottlenecks and genetic drift that degrade the genomes of many endosymbionts [31–35]. With multiple genomes available, mapping of single nucleotide polymorphisms (SNPs) can be used to compare selective pressures on different genes [36, 37] and, together with comparisons of gene repertoire, may show how *Nanoarchaeota* have specialized to different hosts or environmental niches.

In this study, we have analyzed 22 *Nanoarchaeota* SAGs from three hot springs in YNP; some of these *Nanoarchaeota* were co-sorted with their putative hosts, allowing us to suggest expansions to host range. We leveraged single-cell genomics of these co-sorted cells [38] to investigate the diversity of *Nanoarchaeota* on a single host cell. Lastly, we performed SNP analysis to look at patterns of selection within functional categories of genes, using diversification as a signature for proteins potentially involved in symbiosis. Exploring the functions of these proteins allowed us to draw new parallels between terrestrial and marine *Nanoarchaeota,* and with other microbe-microbe symbioses.

## Methods

### Single-cell sequencing and SAG binning

Hot spring sediment samples for single-cell genomics were collected from Cistern Spring and Echinus Geyser hot springs in YNP in 2011 (Additional file 1: Figure S1) [39]. Cells were separated from sediment, concentrated using Nycodenz density gradient centrifugation, and frozen on dry ice. Single cells were isolated using fluorescent-activated cell sorting (FACS), lysed and whole genome amplified with multiple displacement amplification (MDA), and MDA products were screened with 16S rRNA gene PCR according to DOE JGI standard protocols [40]. Based on 16S rRNA gene sequences, 6 *Nanoarchaeota* cells were selected, 2 from Cistern Spring and 4 from Echinus Geyser. Nextera libraries with a target insert size of 300 were sequenced on the Illumina NextSeq platform following the standard Illumina TruSeq protocol (Illumina) generating between 12,722,302 and 23,436,168 reads per SAG (Additional file 2: Table S1). Adapters were trimmed from the sequence data, reads were filtered for quality, errors were corrected with tadpole, and a kmer normalization was performed using bbnorm; the latter two steps were performed with the bbtools package [41]. Filtered reads were then assembled with SPAdes version 3.10.1 [42] with kmer sizes of 25, 55, and 95; resulting scaffolds were trimmed by 200 bp on each end, and trimmed scaffolds greater than 2 kb in length were retained.

We discovered after sequencing that some of the SAGs contained both *Nanoarchaeota* and putative host genomes, and therefore are not technically single amplified genomes. However, these do represent single sorting events, so for the sake of simplicity, we will refer to all MDA products originating from a single sorting event as SAGs. We use “single-sort” to refer to SAGs containing only *Nanoarchaeota* sequence and “co-sort” to refer to SAGs containing both *Nanoarchaeota* and putative host sequence. Bins derived from co-sort SAGs are referred to as genome bins. Co-sorted SAGs were also detected among 16 recently generated SAGs of *Nanoarchaeota* from Nymph Lake in YNP, so these were added to the analysis to expand the survey of putative host range and environments [28, 29]. To separate scaffolds originating from *Nanoarchaeota* and putative hosts within the 6 SAGs from this study and the 16 SAGs from Nymph Lake (Additional file 2: Table S1; Additional file 1: Figure S1), scaffolds were binned using MetaBAT [43] with default settings and a minimum bin size of 50 kb, then bins were manually refined in Anvi’o [44] based on GC content and BLAST [45] comparison to NCBI nr. Standard assembly statistics, completeness, and redundancy of bins were assessed with CheckM [46]. Tetranucleotide frequencies (TNF) were calculated for scaffolds, clustered with principal components analysis (PCA), and plotted in R to visually check binning results.

Genome bins were assigned as putative hosts or *Nanoarchaeota* based on their GC content, the lineage assigned by CheckM, TNF PCA plots, and average nucleotide identity (ANI) to reference genomes of hosts and *Nanoarchaeota*. ANI analysis was performed with pyani with -m ANIb [47, 48] and visualized with the superheat package [49] in R. Genome bins were filtered by different criteria for different analyses (Additional file 2: Table S1). *Nanoarchaeota* or putative host genome bins had to be at least 25 kb in size for inclusion in heatmaps, and both bins had to be at least 25 kb for associating *Nanoarchaeota* with putative hosts. For inclusion in the ribosomal protein-based phylogeny, at least 20% of the sites in the concatenated alignment had to contain information, equivalent to approximately six ribosomal proteins.

### Delineation of *Nanoarchaeota* clades

To delineate clades within the *Nanoarchaeota*, a 3-pronged approach was used: 16S rRNA gene similarity, ribosomal protein-based (RP) phylogeny, and ANI. At least two of the following three criteria had to be met in order for genomes or genome bins to be grouped together into a clade: they had to share at least 98% 16S rRNA gene similarity [50], be each other’s nearest neighbors in the RP tree or be part of a branch containing only members of the same clade, or share at least 95% ANI over at least 20 kb of alignment length. Once initial clades were formed, additional genome bins were added to clades based on 95% ANI, but criteria that were not met could only be due to missing data, not to conflicting data. For example, a genome bin might be placed in a clade even if it did not have a 16S rRNA gene sequence but not if it had a 16S rRNA sequence less than 98% similar to others in the clade. All other genomes and genome bins were left unassociated with any clade (“no clade”).

A phylogeny based on the concatenated alignment of ribosomal proteins (RP), the RP tree, was constructed as described previously [51] with some modifications. Briefly, best-hit sequences from 30 ribosomal protein COGs were identified with hmmsearch (HMMER v3.1b2, [52]) and extracted from genomes and *Nanoarchaeota* genome bins. Three COGs were absent from all genomes (COG088, COG0091, COG0099), and 3 COGs which were represented by only 1 *Nanoarchaeota* genome or genome bin (COG0096, COG00197, COG0255) were not included in the concatenated alignment, for a total of 24 COGs. The species tree was calculated with PhyloBayesMPI [53] CAT+GTR in two chains with ~ 3200 trees per chain; the first 25% of trees in each chain were discarded as burn-in and the chains converged with maxdiff < 0.1. The final tree was visualized and annotated in R with ggtree [54]. Pairwise comparisons of 16S rRNA gene similarity were performed in Jalview [55].

A 16S rRNA gene phylogeny was constructed to compare these newly defined clades to the larger context of phylum *Nanoarchaeota* globally. 16S rRNA sequences from *Nanoarchaeota* genome bins and *Nanoarchaeota* reference genomes were identified based on annotation in IMG or by structural homology search with SSU-align [56]. All 16S rRNA gene sequences (at least 400 nt in length) assigned to phylum *Nanoarchaeota* in SILVA (release 128) were verified by the search and classify feature of the online SINA aligner, comparing the query sequence to up to ten neighbors with at least 75% sequence similarity, and sequences re-assigned to phylum *Nanoarchaeota* were retained [57, 58]. An environmental PCR amplicon dataset and PCR amplicons from sorted single cells were also included, and *Candidatus* Mancarchaeum acidiphilum was selected as an outgroup for rooting the tree. Sequences were aligned with SSU-align [56], masked with the default Archaea mask, and a maximum likelihood (ML) tree was created with IQ-TREE [59, 60] with model TN + R3 and 100 bootstraps. Trees were visualized and annotated in R with package ggtree [54], using metadata from SILVA to assign sequences to habitat types.

### Associating *Nanoarchaeota* with putative hosts

Taxonomy was assigned to putative host genome bins by ANI comparison to other genome bins and publicly available references (Additional file 2: Tables S2 and S3), requiring an ANI of at least 95% over at least 20 kb alignment length. None of the putative host genome bins contained a 16S rRNA gene sequence so these were not compared. Standard assembly statistics, completeness, and redundancy of references were assessed with CheckM [46] and used to annotate ANI heatmaps in R with the package superheat [49]. Alluvial plots showing the distribution of *Nanoarchaeota* clades, associated putative hosts, and sampling sites were drawn in R with the package alluvial [61].

To investigate further possible links between *Nanoarchaeota* and their putative hosts, we searched for recent horizontal gene transfer by aligning all proteins at least 100 amino acids in length from our SAGs to each other and to the NCBI nr database [62]. Alignments were sorted by bitscore to obtain the top 10 overall hits, and hits between *Nanoarchaeota* proteins and their putative host (or vice versa for putative host proteins) were retained. Matches were required to be from the same co-sorted SAG or from the same host-*Nanoarchaeota* pairing (for example, clade 2 *Nanoarchaeota* with *Thermocladium* sp.).

### Clonality of *Nanoarchaeota* associated with a single host cell

Reads from SAGs were used to call SNPs and determine if multiple symbionts with distinct genomes were attached to the same host cell. Briefly, reads were mapped from individual SAGs to the corresponding genome bins with bowtie2 (--very-sensitive, global alignment mode) and alignments were filtered to discard reads with less than 95% identity to the assembly, average read quality of less than 30, map quality of less than 20, and bases with a quality of less than 30. Pysam was used to generate read counts of the four nucleotides at each genomic position. In order to make comparisons between SAGs, all SAGs were down-sampled to 50 mapped reads per site. SNPs were called at a minor allele frequency (MAF) of at least 10% in order to minimize the effect of sequencing errors while maintaining sensitivity to detect true SNPs. Mapping and SNP calling was performed on single-sort *Nanoarchaeota* SAGs and co-sorted SAGs where both genome bins were at least 25 kb in size, except two SAGs for which reads were not available (AB-777-F03, AB-777-O03) (Additional file 2: Table S1). The distribution of SNP density was compared between co-sorted *Nanoarchaeota* genome bins, single-sorted *Nanoarchaeota*, and putative host genome bins using a one-way Wilcoxon rank sum test. Single-sorted *Nanoarchaeota* and putative host genome bins were presumed to represent single cells, so their variance served as a baseline for errors introduced by MDA, sequencing, and assembly.

A simulation was performed to estimate the expected number of SNPs that would be observed from multiple distinct *Nanoarchaeota* attached to the same host cell. For this analysis, we selected 14 *Nanoarchaeota* SAGs from Nymph Lake since these symbionts were found in the same environment and therefore are most likely to co-occur on the same host cell. AB-777-F03, the most contiguous large assembly from Nymph Lake (Table 1), was used as a reference for mapping SAG reads, using the same parameters as before. We retained 6 SAGs which covered the reference genome by > 25% (Additional file 2: Table S1). To simulate the presence of multiple attached cells, we pooled mapped reads from between 1 to 6 *Nanoarchaeota* SAGs and used the pooled reads to call SNPs. To equalize differences in sequencing depth, we used the same number of reads from each SAG per genomic position. Each genomic position was down-sampled to 50 mapped reads and SNPs were called at a MAF of at least 10%.

**Table 1:** Assembly statistics, completeness and contamination estimates, and additional information for *Nanoarchaeota* genome bins

Genome bin ID	Assembly size (bp)	# Scaffolds	Longest scaffold (bp)	GC (%)	# Predicted genes	Estimated completeness (CheckM) (%)	Estimated contamination (CheckM) (%)	# Ribosomal proteins (of 24)	Genome quality(MISAG)
AB-777-F03 Nano	449,376	20	95,961	24.5	527	54.67	0	21	Medium
AB-777-O03 Nano	549,214	47	44,534	24.1	656	65.03	6.54	21	Medium
AD-903-B02 Nano	135,497	24	20,845	24.5	184	16.74	0.47	1	Low
AD-903-B22 Nano	218,763	37	15,769	24.3	266	18.54	0	6	Low
AD-903-D09 Nano	28,158	6	11,989	25.2	37	1.25	0	1	Low
AD-903-D23 Nano	101,465	22	17,152	25.8	137	20.91	0	11	Low
AD-903-F05 Nano	125,027	23	12,408	25.1	167	19.63	0	7	Low
AD-903-F18 Nano	56,402	12	9231	23.9	80	12.31	0	8	Low
AD-903-I14 Nano	125,613	25	10,234	25	164	15.29	0.93	6	Low
AD-903-L04 Nano	105,782	15	17,449	26.1	126	10.75	0	1	Low
AD-903-M20 Nano	105,795	21	12,044	25.1	139	19.16	0	6	Low
AD-903-N05 Nano	210,845	37	19,172	24.9	249	26.01	0	4	Low
AD-903-P15 Nano	273,481	40	24,686	24.3	336	34.97	0	5	Low
AD-903-P16 Nano	192,530	37	14,587	25.7	248	29.55	0	11	Low
CS1 Nano	106,721	18	14,946	24.1	136	13.92	0	3	Low
CS2 Nano	199,134	39	16,387	24.2	245	26.87	0	8	Low
EG1 Nano	231,923	36	31,901	25.4	277	17.63	0	4	Low
EG2 Nano	144,926	23	15,304	25.1	179	22.27	0	7	Low
EG3 Nano	65,600	12	10,727	25.6	80	9.06	0	2	Low
EG4 Nano	56,920	15	12,800	25.6	80	8.09	0	3	Low

### Population diversity of *Nanoarchaeota*

We used the tool MIDAS [63] to investigate the diversity of *Nanoarchaeota* within a single population using the same mapping and filtering parameters as before. Specifically, we used SAG reads from clade 1 SAGs from Nymph Lake, the clade with the largest number of representatives. Reads from SAGs with clade 1 *Nanoarchaeota* genome bins at least 100 kb in size (*n* = 7, Additional file 2: Table S1) were mapped against AB-777-F03, as described above. Synthetic reads were created for AB-777-O03 by shredding contigs with randomreads.sh from the bbtools package [41] with 20X coverage, insert size range of 180–400 nt, read length of 150 nt, and without simulating sequencing error. To minimize the effect of sequencing errors, we used mapped reads to call the consensus allele at each genomic position within each SAG and masked sites where > 10% of the reads differed from each other. SNPs were called at genomic positions covered by at least 5 of the 7 SAGs where at least 1 SAG had an observed variant relative to the other SAGs or the reference. Within protein coding regions, we identified SNPs at fourfold degenerate sites (i.e., synonymous SNPs or sSNPs) and SNPs at onefold degenerate sites (i.e., non-synonymous SNPs or nSNPs). As a measure of selective pressure, we computed pN/pS, defined as the ratio of the number of nSNPs per non-synonymous site to the number of sSNPs per synonymous site. SNP density was computed genome wide, for classes of SNPs and for individual genes. Genes were divided into functional categories based on their annotations in IMG and only genes with at least 100 total mapped sites were considered. SNP density was compared between functional categories with at least ten genes (excluding categories oxidative stress, secretion, and transporters) using a one-way analysis of variance and post hoc Tukey HSD test in R. For two proteins with high nSNP densities (see the “Results and discussion” section), we tested whether the distribution of nSNPs (amino acid substitutions) between internal, external, and transmembrane regions of the proteins was significantly different with chi-square tests on alignments trimmed and divided in R.

## Results and discussion

### Diversity and clades within *Nanoarchaeota*

In this study, we utilized single-cell genomics to address ecological and evolutionary questions about *Nanoarchaeota* and their hosts that could not be tackled by previous studies focusing on single examples of associations. From a total pool of 22 SAGs, 4 of the 6 SAGs sequenced in this study and 6 of the 16 SAGs from Nymph Lake [28, 29] represented co-sorted SAGs with both *Nanoarchaeota* and putative host genome bins larger than 25 kb (Table 1, Additional file 2: Tables S1 and S4). *Nanoarchaeota* genome bins ranged from 28,158 bp to 549,214 bp in size (Table 1) and were clearly separated from putative host genome bins by TNF PCA in all co-sorted SAGs (Additional file 1: Figure S2). These symbiont bin sizes approximated 1 to 83% estimated genome completeness (Table 1, Additional file 2: Table S4). Although this is less than observed for single-cell genomes of benchmark cultures [64], the low estimates can be explained by low sequencing coverage of some SAGs (Additional file 1: Table S1). Additionally, the absence of some standard single-copy marker genes from *Nanoarchaeota* results in estimated completeness values lower than actual genome completeness. Overall, 2 *Nanoarchaeota* and 3 putative host genome bins met medium-quality draft MISAG standards [65], all others were low-quality drafts.

**Fig. 1:**
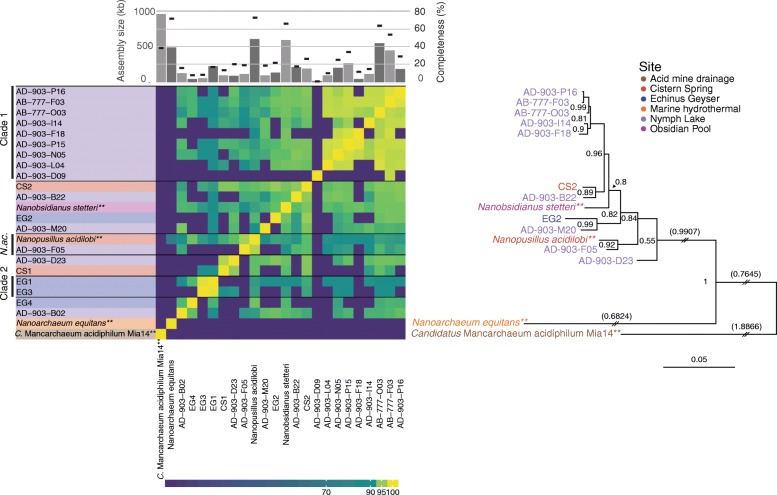
Genome-based phylogeny of phylum *Nanoarchaeota.* Heatmap of ANI, with Bayesian phylogeny based on the concatenated alignment of ribosomal proteins. ANI comparisons with alignment lengths less than 20 kb were set to 0% ANI. Only genomes with information in at least 20% of aligned sites were included in the phylogenetic tree, omitting genomes with insufficient information. Species-level clades derived from a combination of ANI, 16S rRNA gene similarity, and Bayesian phylogeny are delineated by black bars beside SAG or genome names. Bar labeled “*N.ac.*” denotes a clade formed by the cultivated species *Nanopusillus acidilobi* and a SAG. Clade 2 was derived from ANI and 16S rRNA gene similarity only; these genomes had insufficient ribosomal protein information for inclusion in the phylogeny. Genomes are colored by sampling location, and ** indicates a publicly available reference genome. Assembly size (bars) and estimated completeness (dashes) of *Nanoarchaeota* genomes are shown above the heatmap. Note that the reduced genomes of *Nanoarchaeota* result in estimated completeness < 100% even for closed genomes (e.g., *N. equitans*). Branches in the tree with a double slash have been re-scaled, and their actual branch lengths are shown in parentheses. Branch support values are indicated at tree nodes. *Candidatus* Mancarchaeum acidiphilum is included as an outgroup and is not part of phylum *Nanoarchaeota*

Results from ANI analysis, 16S rRNA gene similarity, and ribosomal protein phylogeny defined *Nanoarchaeota* groupings that were consistent for all cases where multiple metrics were available (Fig. 1, Additional file 2: Tables S5, S6, and S7). Based on these metrics, two novel approximately species-level clades of *Nanoarchaeota* were identified with 95–98% 16S rRNA gene similarity and 88.7–91.8% ANI to each other and to described species of *Nanoarchaeota*. These clades were used as a foundation for subsequent analyses. Clade 1 contained 9 genome bins from Nymph Lake, clade 2 contained 2 genome bins from Echinus Geyser, and 1 genome bin was associated with the previously described species *Nanopusillus acidilobi*. Eight genome bins could not be grouped into clades with other genome bins or references (Additional file 2: Table S1). Clades were restricted to single sampling locations, with the exception of *N. acidilobi* which we found in Nymph Lake (AD-903-F05) and was observed previously in Cistern Spring (Fig. 1). Cistern Spring, Echinus Geyser, and Nymph Lake all harbored multiple clades of *Nanoarchaeota* (Fig. 1).

There are numerous members of the phylum *Nanoarchaeota* with 16S rRNA gene sequences that are only about 80% similar to those from sequenced genomes (Additional file 1: Figure S3); for example, *N. equitans* has 82.1% (± 0.42) mean 16S rRNA gene similarity to other full-length sequences (Additional file 2: Table S7). Even near-identical 16S rRNA sequences can accompany very different genome content [66, 67], thus considering only 16S rRNA gene sequences can mask extensive genetic diversity and niche partitioning. The majority of available *Nanoarchaeota* 16S rRNA gene sequences and sequenced genomes originated from hot springs within YNP, but much of the diversity within the phylum is found in hydrothermal sediment, marine, and hypersaline habitats and is still not represented by sequenced genomes, or even full-length 16S rRNA gene sequences (Additional file 1: Figure S3). These *Nanoarchaeota* without genomic representation are likely to encode functional diversity critical for biogeochemical processes and evolutionary diversification of microorganisms within these ecosystems.

### Associating *Nanoarchaeota* with putative hosts

**Fig. 2:**
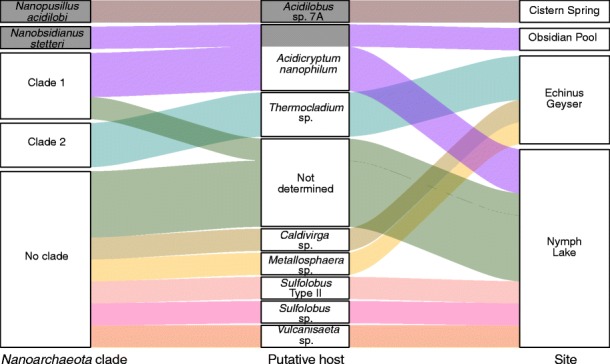
Association of terrestrial *Nanoarchaeota*, known or putative hosts, and sampling sites. *Nanoarchaeota* clades are as shown in Fig. 1, including 3 SAGs from clade 1, 2 SAGs from clade 2, and 8 SAGs not assigned to a clade. ANI identification of putative hosts is shown in Additional file 1: Figure S4. Putative hosts listed as “Not determined” shared less than 95% ANI and/or less than 20 kb aligned length with all other genome bins and references. Only references and SAGs where both *Nanoarchaeota* and putative host genome bins were at least 25 kb in size are shown. Connections are colored by putative host, and known symbioses are shaded in gray

Co-sorting of *Nanoarchaeota* attached to other cells has been observed previously [28], and these have been experimentally demonstrated to be host cells [16, 17]. In this study, we expanded on this by investigating a large number of co-sorted cells from multiple sampling sites to identify novel putative hosts. Taxonomy was assigned to putative host genome bins in 9 of 10 co-sorted SAGs based on ANI to references (Additional file 1: Figure S4; Additional file 2: Table S8). Seven putative hosts were associated with *Nanoarchaeota* genome bins (Fig. 2), including the previously known host Acd1 “Acidicryptum nanophilum,” which was observed with *Nanoarchaeota* clade 1 in two SAGs, lending support that the co-sorting method recovers genuine biological associations. Our data suggests that three other members of the Order *Sulfolobales* were hosts: *Metallosphaera* sp., *Sulfolobus* type II, and *Sulfolobus* sp*. Thermocladium* sp., *Caldivirga* sp., and *Vulcanisaeta* sp. are the first members of Order *Thermoproteales* implicated as possible hosts. Co-occurrence data from Kamchatka hot springs previously suggested but could not confirm *Vulcanisaeta* as a host [24]. Clade 2 *Nanoarchaeota* were found to associate with *Thermocladium* sp. in two SAGs. Four proteins were found to likely be horizontally transferred between *Thermocladium* sp. and clade 2 *Nanoarchaeota* (Additional file 2: Table S9). All lacked functional annotation but may provide useful information for future studies. Each *Nanoarchaeota*-putative host pairing was restricted to a single sampling site, although Nymph Lake and Echinus Geyser harbored multiple host-symbiont pairs (Fig. 2).

Notably, our data also suggest an expansion of the possible associations for the host “A. nanophilum”. Previous samples from the Obsidian Pool found this host with “Nanobsidianus stetteri” [17, 24], whereas at Nymph Lake it was associated with the closely related clade 1 *Nanoarchaeota* (Fig. 2). This new diversity of putative host-symbiont associations raises questions about their molecular mechanisms of attachment and metabolite transfer. The physical interface between *N. equitans* and *I. hospitalis* is complex, presumably due to the unique anatomy of *I. hospitalis* [2, 9]. The connections between terrestrial *Nanoarchaeota* and their hosts have not been visualized at this level of detail, but are likely to differ substantially from those of *N. equitans,* based on what is known about the morphology and physiology of their hosts. The known and putative hosts of terrestrial *Nanoarchaeota* have a cell envelope consisting of an S-layer protein or proteins [68, 69], whereas in *I. hospitalis* the S-layer is absent [2]. Further, the detailed architecture of the S layer is conserved between some putative hosts such as those within Order *Sulfolobales* [68]. These factors indicate that the mechanisms and structures that mediate host-symbiont interactions in terrestrial *Nanoarchaeota* may be more generalized or perhaps more rapidly evolving, facilitating a broader host range.

### Clonality of *Nanoarchaeota* associated with a single host cell

Multiple *Nanoarchaeota* cells are commonly observed attached to a single host cell, both in culture and in situ [5, 16, 28, 70]. However, it is not known if these cells are clonal or if the attachment of multiple, diverse *Nanoarchaeota* cells is possible. To assess this, we used read-mapping to quantify the density of SNPs in genome bins of attached (co-sorted) *Nanoarchaeota*. As a control, we compared this to SNPs found in genome bins of *Nanoarchaeota* putative hosts and unattached (single-sort) *Nanoarchaeota* (Additional file 2: Table S1). Putative hosts were expected to be single cells, because doublets of these larger cells would have been too large to be included within the FACS gating. Likewise, single-sort *Nanoarchaeota* were also expected to be single cells, as has been observed in culture. Both of these groups served as a baseline for the number of SNPs to expect from a single cell. If the number of SNPs in co-sorted *Nanoarchaeota* was similar to this baseline, we expect that either only a single symbiont cell was attached to a host cell, or all of the symbiont cells arose from the proliferation of the same parent cell. If the number of SNPs in co-sorted *Nanoarchaeota* was significantly greater than the baseline, it would be an indication that there were multiple, different *Nanoarchaeota* attached to the same host cell.

**Fig. 3:**
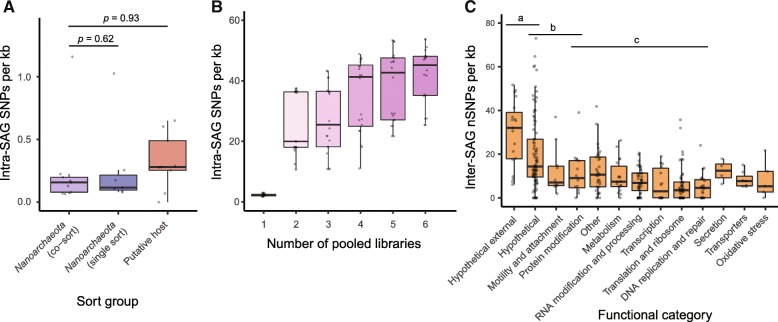
Diversity of *Nanoarchaeota* on a single host cell and within a population. **a** SNPs per kb (MAF > 10%) in single-sorted *Nanoarchaeota* SAGs, co-sorted *Nanoarchaeota* genome bins, and co-sorted putative host genome bins. There was no significant difference (one-way Wilcoxon rank sum tests) between categories. **b** SNPs per kb (MAF > 10%) in pooled datasets of 1 to 6 SAGs, simulating results expected if multiple distinct *Nanoarchaeota* were attached to a host cell. **c** Variation in nSNP density in clade 1 *Nanoarchaeota* genes, summarized by functional category. Different letter groups have significantly different nSNP densities, from ANOVA (one-way ANOVA, *F*(9, 297) = 12.66, *p* < 2e−16) and post hoc Tukey HSD tests (*p* < 0.05) (Additional file 2: Table S13). No bar over boxes indicates categories with fewer than ten genes which were not included in ANOVA or post hoc tests

We found no significant difference in SNP density with putative hosts (one-way Wilcoxon rank sum test, *p* = 0.93) or with single-sort *Nanoarchaeota* (one-way Wilcoxon rank sum test, *p* = 0.62) (Fig. 3a; Additional file 2: Table S10). The 0.25 SNPs per kb observed for co-sorted *Nanoarchaeota* is likely caused by a combination of errors from amplification, sequencing, and assembly [64, 71]. As an additional control, we pooled reads from multiple *Nanoarchaeota* SAGs to simulate diverse populations and estimate the number of SNPs expected from multiple attached cells. Here, we observed a range of 10–50 SNPs per kb when pooling reads from between 2 and 6 SAGs (Fig. 3b, Additional file 2: Table S11). Together, these results indicate that multiple diverse *Nanoarchaeota* were not attached to individual host cells—instead, there were either multiple clonal cells or only one cell per host. Although we cannot rule out the latter possibility, it seems unlikely given extensive imaging of marine and terrestrial *Nanoarchaeota* co-cultures [1, 5, 16] and environmental samples [28]. *Nanoarchaeota* are only capable of dividing while attached to a host, and often appear in clustered or linear arrangements (Additional file 1: Figure S5) that are consistent with the proliferation of a single symbiont. We suggest that future studies should leverage single-cell genomics in combination with imaging to determine the patterns of symbiont abundance on a host in situ, and further elucidate the molecular underpinnings of host establishment and the exclusivity of the symbiont in a given host.

### Fine-scale genomic diversity of *Nanoarchaeota*

To assess genomic variation on a population scale, we analyzed the diversity within the population of clade 1 *Nanoarchaeota* from Nymph Lake. This was the largest species-level group of SAGs in our dataset and was sampled from the same location and date, so they represent a snapshot of diversity within this population. Genome wide, we observed an average of 28 SNPs per kb indicating significant levels of diversity within the clade. Additionally, the pN/pS ratio of 0.159 suggests strong purifying selection and large population size. This pattern was also consistent when comparing individual SAGs to the reference genome (Additional file 1: Figure S6). There were relatively few SNPs in intergenic regions (Additional file 1: Figure S6; Additional file 2: Table S12), likely due to the high coding density in *Nanoarchaeota*. These patterns contrast with those of many microbial endosymbionts, which are subject to population bottlenecks, isolation, and genetic drift, eventually reaching “genomic stasis” [72–74]. Several factors act against these restrictions in *Nanoarchaeota:* they may be motile at some stage in their life cycle [17], they are externally attached to their hosts, and they are often abundant and diverse in situ [12, 28, 29]. We observed considerable diversity even within this small sampling of clade 1 genome bins. Thus, we postulate that population bottlenecks are much less severe in *Nanoarchaeota* than in endosymbionts and that selection rather than genetic drift is primarily responsible for fixing mutations in *Nanoarchaeota.*

Given this pattern of purifying selection, we expected to find different densities of nSNPs across different functional categories of genes, depending on how essential the genes are and if they are involved in interaction with a host. Indeed, while there were no significant differences in the densities of sSNPs across functional categories of genes (one-way ANOVA, *F*(9, 297) = 0.989, *p* = 0.449) (Additional file 2: Figure S7), some functional categories did have significantly different densities of nSNPs (one-way ANOVA, *F*(9, 297) = 12.66, *p* < 2e−16) (Fig. 3c; Additional file 2: Table S13). Highly essential categories such as translation and ribosome proteins, DNA replication and repair, RNA modification and processing, and transcription expected to be under strong selective pressure to maintain function had low densities of nSNPs (Fig. 3c). A few categories had significantly higher densities of nSNPs, including protein modification, motility and attachment, hypothetical proteins, and hypothetical proteins predicted to be cell surface-exposed (based on the presence of a single transmembrane helix motif near the N-terminus of the protein, “Hypothetical external” category) (Fig. 3c; Additional file 2: Table S13). The importance of cell surface modification is apparent in *N. acidilobi* where 10% of the proteome is likely involved in glycosylation of the cell surface [16]. Some of the external proteins with high SNP densities may be involved in detecting and binding to hosts or evading host defenses, as shown in other host-symbiont partnerships [75–77], and rapid evolution is frequently observed in these proteins [77, 78]. Notably, even in an insect endosymbiont with extremely low diversity, cell surface proteins are among the genes with the greatest number of SNPs and other variants [78].

### Diversification of proteins involved in symbiosis

**Fig. 4:**
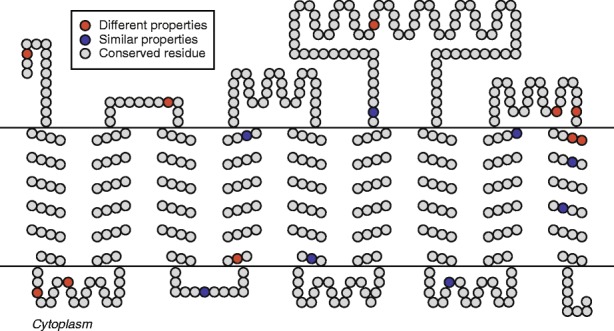
nSNPs in cytochrome bd-I ubiquinol oxidase in clade 1 *Nanoarchaeota*. Cartoon of cytochrome bd-I ubiquinol oxidase with residues with nSNPs highlighted, based on the properties of the alternate residue(s) compared to the reference residue in AB-777-F03. sSNPs in conserved residues are not shown

Some of the genes with the highest densities of nSNPs may help explain our findings of a potentially broad host range, clonality on a single host, and purifying selection in the overall population. One of these genes is cytochrome bd-I ubiquinol oxidase subunit I (IMG Gene ID 2735310658, Additional file 3), one subunit of a membrane-bound enzyme which transfers electrons from a reduced quinol to O_2_, generating membrane potential without pumping protons [16, 79, 80]. Subunit II of this enzyme was not annotated by an automated pipeline, but this subunit often has a faster evolutionary rate than subunit I and this divergence can prevent automatic annotation of homologues [81]. Immediately downstream of subunit I, we found a hypothetical integral membrane protein which displays distant similarity (~ 25% amino acid identity) to subunit II. Within subunit I, there was no significant difference in the distribution of nSNPs between different regions (internal, external, transmembrane) (χ ^2^ (2, *N* = 453) = 0.31574, *p* = 0.854). The functionally important Q-loop responsible for binding with the O_2_ substrate was conserved except for two nSNPs (Fig. 4), leading us to postulate that it is still a functional enzyme. However, we found neither the ability to synthesize any quinones nor the ability to reduce quinones to quinols, the substrates for this enzyme, within any terrestrial *Nanoarchaeota* genomes. *Nanoarchaeota* may be utilizing reduced quinols from the host diffusing through membranes [79] where the cells are connected, and nSNPs in the transmembrane regions of the oxidase might allow them to utilize different quinols if they are associated with different hosts (Fig. 4).

Quinone-mediated energy transfer has been experimentally demonstrated in *Geobacter* [82], and evidence from other microbe-microbe symbioses suggests this may be a common mechanism for energy exchange. “*Chlorochromatium aggregatum*” is proposed to exchange quinones between the bacterial partners to create a shared proton motive force to power motility of the central bacterium [75]. Multiple *Parcubacteria*, also believed to rely at least partially on other microbes, have ubiquinol oxidases but no quinone biosynthesis genes or quinone-dependent NAD(P)H dehydrogenases [76]. *N. equitans* is an interesting exception from the terrestrial representatives of the phylum in that it possesses a sulfide:quinone oxidoreductase, but not a cytochrome bd-I ubiquinol oxidase. Multi-omics studies have suggested that *N. equitans* may use NADH from *I. hospitalis* with this oxidoreductase to generate ATP [8]. If true, *N. equitans* may be utilizing a similar strategy to that proposed for terrestrial *Nanoarchaeota* but drawing from a different step on the host electron transport chain. *Candidatus* Mancarchaeum acidiphilum Mia14 has both a sulfide:quinone oxidoreductase (IMG Gene ID 2758411520) and a cytochrome bd-I ubiquinol oxidase (IMG Gene IDs 2758412059, 2758412060) [22], so it could be utilizing either strategy.

We speculate that quinone-mediated energy transfer between *Nanoarchaeota* and hosts could lead to clonality on a host. In this proposed scenario, an attached *Nanoarchaeota* cell would use reduced quinols from the host and thereby slightly deplete the host membrane potential. High membrane potential might be required for successful attachment to a host or to obtain the necessary energy to proliferate on a host. In such a case, other *Nanoarchaeota* encountering an occupied host cell might fail to initiate or complete attachment due to this reduction in membrane potential. Thus, only the progeny of the first symbiont to attach would proliferate on an individual host cell. Such a process could also apply to *N. equitans*. Unlike other known prokaryotes with two membranes, *I. hospitalis* has a charged outer membrane due to the localization of ATP synthase in this layer [83], so it would be theoretically possible for *N. equitans* to sense the membrane potential. The clustering of *Nanoarchaeota* cells on a host cell commonly observed in cultures of both *N. equitans* [1, 5] and *N. acidilobi* [16] is consistent with this proposed mechanism. It is also possible that clonality could arise as a consequence of the spatial separation of potential hosts, such that the probability of a given symbiont cell encountering and attaching to a host is low, and the probability of two different symbiont cells attaching to the same host is even lower. Single-cell genomics could be used to determine if clonality is the exception or the rule in other microbe-microbe associations where multiple ectosymbionts are observed attached to a single host. These include TM7 (Saccharibacteria) [25], *Candidatus* Mancarchaeum acidiphilum Mia14 [22], and phototrophic consortia like “*Chlorochromatium aggregatum*” [84]. The latter is an especially interesting point of comparison as the ectosymbionts appear to be vertically transmitted when the central cell divides [75].

The second gene with a high density of nSNPs and possible function in the symbiosis is from the “Motility and attachment” functional category. It was annotated as a type 2 secretion system (T2SS) protein F, homologous to FlaJ/TadC, a membrane platform protein for secretion systems, pili, and flagella [85] (IMG Gene ID 2735310502). There was no significant difference in the distribution of nSNPs between internal, external, and transmembrane regions of the protein (***χ***^2^ (2, *N* = 619) = 2.2621, *p* = 0.3227). T2SS have many similarities to type IV pili (T4P) [85] which are abundant and diverse in Archaea [86], so this gene likely encodes part of a T4P system. Genomic, proteomic, and microscopy data indicate that *Nanoarchaeota* have two different T4P systems as well as a true archaeal flagellum [16, 70, 86]. These appendages likely serve different purposes and can be regulated separately [86]. The flagellum commonly plays a role in motility and generalized attachment to surfaces and other cells [87] and may do the same in *Nanoarchaeota* and other microbial symbionts [88–90]. Once *Nanoarchaeota* have formed a weak non-specific attachment to a potential host with the flagellum, the T4P systems might be responsible for sensing the suitability of the host and forming a more secure and specific attachment. Thus, diversification of the T4P systems could be indicative of adaptation to different hosts or compensatory mutations to escape host defenses. The presence of multiple T4P systems broadens this adaptive potential, and interestingly, we found four different FlaJ/TadC homologues with densities of nSNPs from 1.9–37.0 per kb (Additional file 3), suggesting varying levels of evolutionary pressure on these different systems. The importance of T4P and pili in general is apparent by their ubiquity in other microbial symbionts, even those with highly reduced genomes and missing many key metabolic functions [22, 23, 76, 91–93].

In this study, single-cell genomics has enabled us to perform a detailed genomic analysis and identify genes that are diversified in a *Nanoarchaoeota* population and with putative roles in symbiosis. Several of these genes have been repeatedly implicated in host association in other symbioses, lending validity to our approach and conclusions. However, there are important differences in the life history, population diversity, and genomic signatures of selection in *Nanoarchaeota* compared to microbial endosymbionts of eukaryotes [34, 94]. This suggests that additional comparison with other microbe-microbe symbioses is needed to clarify which molecular mechanisms underpin these types of associations, what genes and proteins influence host range and host switching, and to what degree are they diverged or conserved. Importantly, *Nanoarchaeota* are only a single lineage within the DPANN superphylum, many of which are also known or hypothesized to depend on a microbial host [21–23], as are many members of an analogous group of bacteria, the Candidate Phyla Radiation (CPR, or superphylum Patescibacteria) [92, 95]. Thus, experimentally tractable *Nanoarchaeota*-host systems may be able to shed light on the molecular mechanisms of microbe-microbe association that could be shared across large swaths of the tree of life.

## Conclusions

In summary, we have demonstrated that *Nanoarchaeota* can be readily co-sorted with putative hosts in a high-throughput and culture-independent manner using single-cell genomics techniques, enabling us to perform a detailed genomic analysis. Using these data, we have derived a genome-based phylogeny of *Nanoarchaeota*, defining two species-level clades and suggesting that global diversity remains greatly underexplored. Six novel putative hosts for YNP *Nanoarchaeota* are proposed, and we suggest that the known host Acd1 "Acidicryptum nanophilum" can associate with multiple species of *Nanoarchaeota*, broadening the range of possible associations for both hosts and symbionts. Single-cell genomics of co-sorted associations also allowed us to determine that although populations of *Nanoarchaeota* are diverse, those attached to a single host cell appear to be multiple clonal cells or present as single cells. High overall SNP densities and a low pN/pS imply purifying selection and important differences in evolutionary processes compared to obligate microbial endosymbionts. Genes with high densities of nSNPs included likely cell surface proteins, type IV pili components, and a cytochrome bd-I ubiquinol oxidase, all of which are implicated in interactions with hosts in other microbial symbioses. Based on these genes, we propose a hypothesis for how clonality may be maintained in this symbiosis. Together, these results provide clues about the adaptation of *Nanoarchaeota* to such a broad range of potential hosts and environmental conditions, providing a new foundation for our understanding of the many other microbe-microbe symbioses thought to exist within the major, yet largely uncultivated branches of the tree of life.

## Additional files

Additional file 1: **Figure S1.** Map of sampling sites. **Figure S2.** TNF PCA plots for SAGs illustrating separation of *Nanoarchaeota* and putative host genome bins. **Figure S3.** Maximum likelihood phylogeny of phylum *Nanoarchaeota* based on 16S rRNA gene sequences at least 400 nt in length. **Figure S4.** Identification of putative host genome bins based on ANI to reference genomes and metagenome bins. **Figure S5.** Scanning electron micrograph of multiple *Nanoarchaeota* cells attached to host cells. **Figure S6.** SNP type and density in individual clade 1 *Nanoarchaeota* SAGs. **Figure S7.** Variation in sSNP density in clade 1 *Nanoarchaeota* genes by functional category. (DOCX 24585 kb)

Additional file 2: **Table S1.** Read count, assembly statistics, completeness and contamination estimates from CheckM, and *Nanoarchaeota-*host pairing information for SAGs and selected *Nanoarchaeota* reference genomes. **Table S2.** Data identifiers for SAGs and reference genomes used in this study. **Table S3.** Assembly statistics, completeness and contamination estimates from CheckM, and additional information for reference genomes for comparison to putative host genome bins. **Table S4.** Assembly statistics, completeness and contamination estimates from CheckM, and additional information for *Nanoarchaeota* genome bins < 25 kb, putative host genome bins, and unbinned scaffolds. **Table S5.** Congruence of 16S rRNA gene, ribosomal protein tree, and ANI data for delineating *Nanoarchaeota* genome bins into clades. **Table S6.** Average nucleotide identity (ANI) of *Nanoarchaeota* genome bins and reference genomes. Comparisons > 95% ANI are highlighted. All cells with alignment lengths less than 20 kb have been set to 0% ANI. **Table S7.** Similarity of *Nanoarchaeota* 16S rRNA gene sequences extracted from genome bins and references. Sequences that are > 98% similar are highlighted in green, sequence lengths are shown in parentheses. **Table S8.** Average nucleotide identity (ANI) of putative host genome bins and host reference genomes. Comparisons > 95% ANI are highlighted. All cells with alignment lengths less than 20 kb have been set to 0% ANI. **Table S9.** Alignment results for proteins potentially horizontally transferred between hosts and *Nanoarchaeota*. **Table S10.** Within-SAG SNPs in single-sorted *Nanoarchaeota* SAGs and co-sorted *Nanoarchaeota* and putative host genome bins, at a MAF > 10%. **Table S11.** Simulated within-SAG SNPs for pooled datasets of 1 to 6 SAGs, at a MAF > 10%. **Table S12.** SNPs in all clade 1 *Nanoarchaeota* genome bins (> 100 kb bin size), mean values shown for each site type. **Table S13.** Pairwise comparisons of mean non-synonymous SNPs per kb between functional categories of genes in clade 1 *Nanoarchaeota* SAGs and SAG bins, from post-hoc Tukey HSD tests. Significant comparisons are highlighted in green. (XLSX 65 kb)

Additional file 3: sSNP and nSNP data for genes in clade 1 *Nanoarchaeota* SAGs and genome bins, organized in tabs by functional category. Genes highlighted in the discussion are separated into their own tab and highlighted in green within their respective functional category tabs. (XLSX 119 kb)

## Abbreviations

ANI: Average nucleotide identity
DPANN: Superphylum of Archaea originally comprising Diapherotrites, Parvarchaeota, Aenigmarchaeota, Nanohaloarchaeota, and Nanoarchaeota, now including also Woesearchaeota, Pacearchaeota, and Micrarchaeota
MAF: Minor allele frequency
MDA: Multiple displacement amplification
ML: Maximum likelihood
nSNP: Non-synonymous SNP
PCA: Principal component analysis
pN/pS: Ratio of non-synonymous SNP/non-synonymous site to synonymous SNP/synonymous site
RP: Ribosomal protein
SAG: Single amplified genome
SNP: Single nucleotide polymorphism
sSNP: Synonymous SNP
TNF: Tetranucelotide frequency
YNP: Yellowstone National Park
