# Supplementary material for: Single-cell genomics of co-sorted Nanoarchaeota suggests novel putative host associations and diversification of proteins involved in symbiosis
Source: Microbiome. 2018 Sep 17;6:161. doi: 10.1186/s40168-018-0539-8 (PMC6142677; doi:10.1186/s40168-018-0539-8)
Supplement: Supplementary file 1 — Figure S1. Map of sampling sites. Figure S2. TNF PCA plots for SAGs illustrating separation of Nanoarchaeota and putative host genome bins. Figure S3. Maximum likelihood phylogeny of phylum Nanoarchaeota based on 16S rRNA gene sequences at least 400 nt in length. Figure S4. Identification of putative host genome bins based on ANI to reference genomes and metagenome bins. Figure S5. Scanning electron micrograph of multiple Nanoarchaeota cells attached to host cells. Figure S6. SNP type and density in individual clade 1 Nanoarchaeota SAGs. Figure S7. Variation in sSNP density in clade 1 Nanoarchaeota genes by functional category. (DOCX 24585 kb) [file 40168_2018_539_MOESM1_ESM.docx]

**
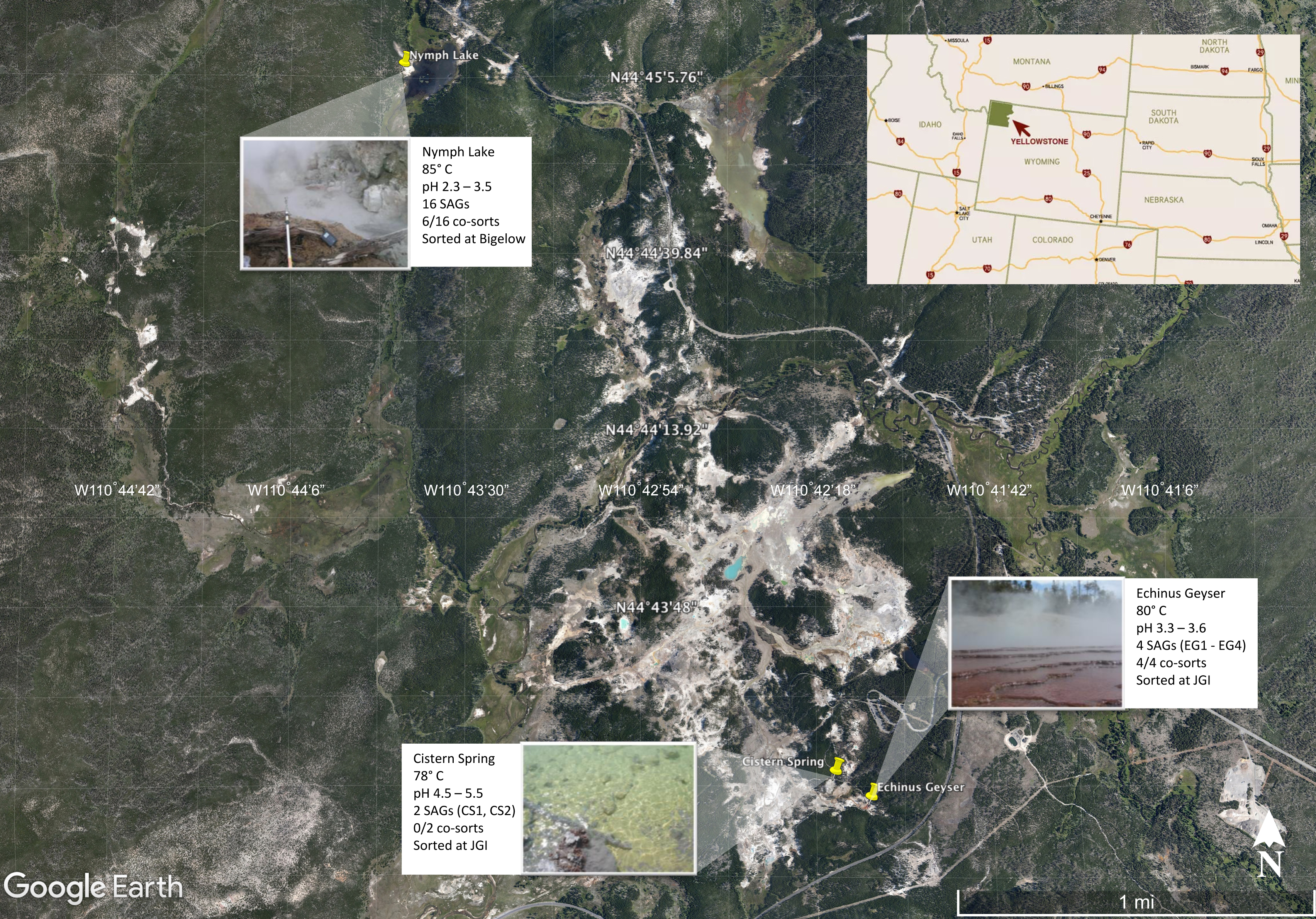
**

**Figure S1. Map of sampling sites.** Locations and photographs of sampling sites with environmental metadata, total number of SAGs and number of co-sorted SAGs from each site.


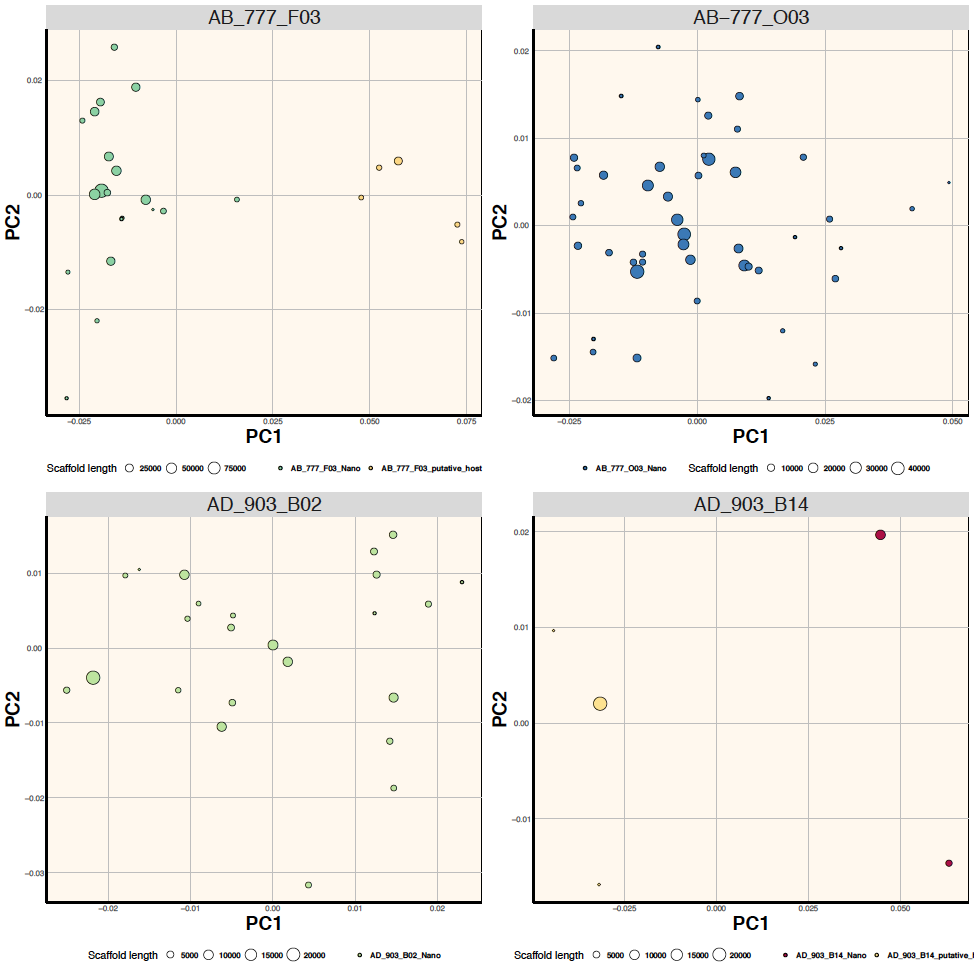


**
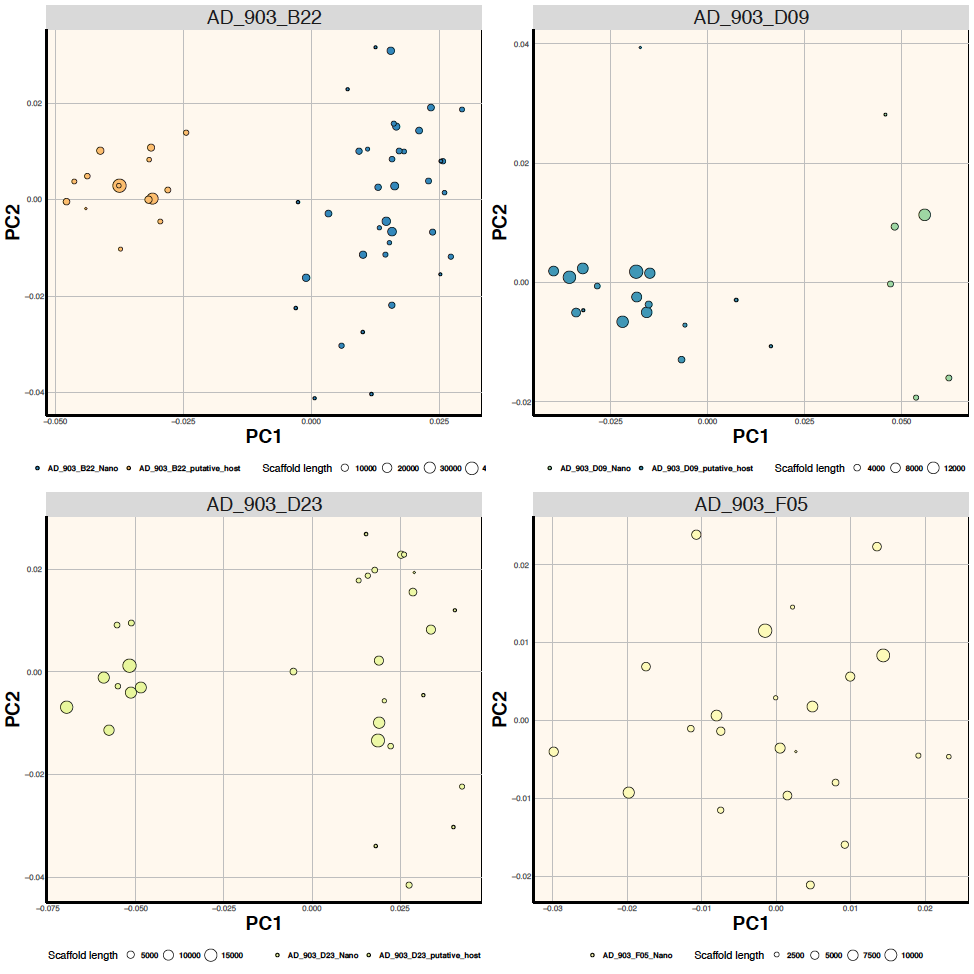
**

**
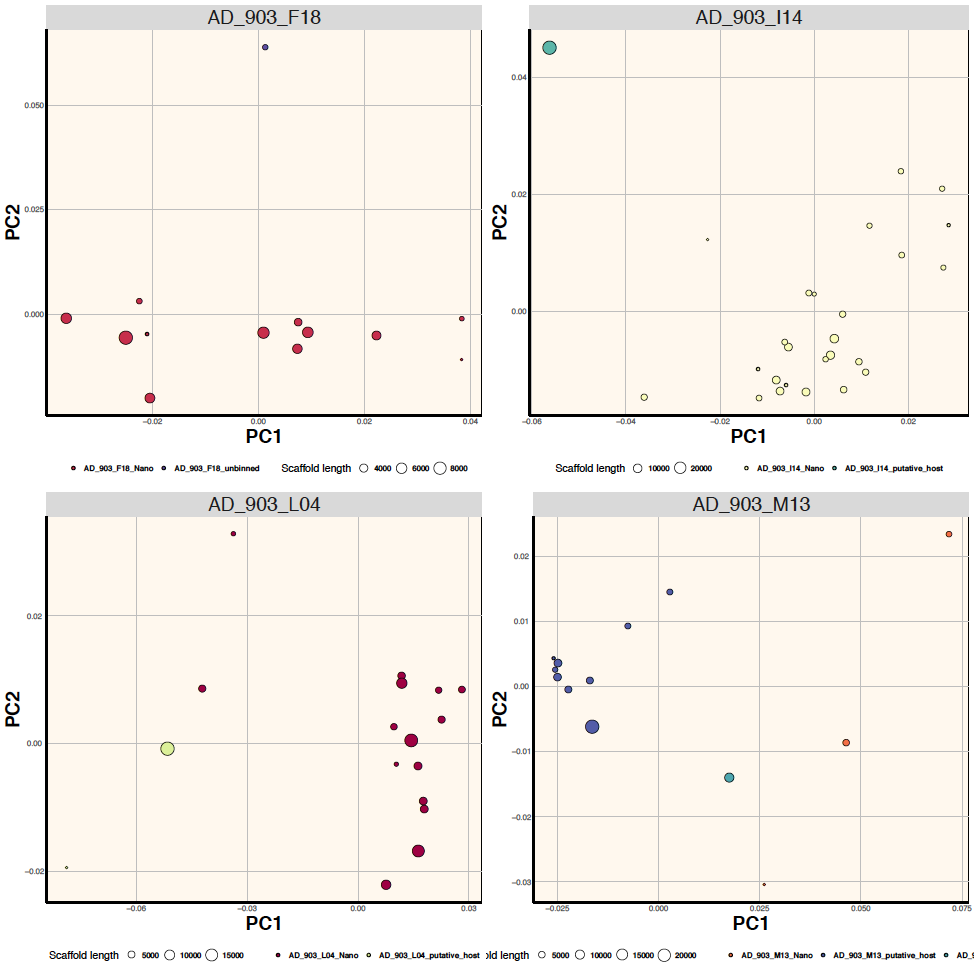
**

**
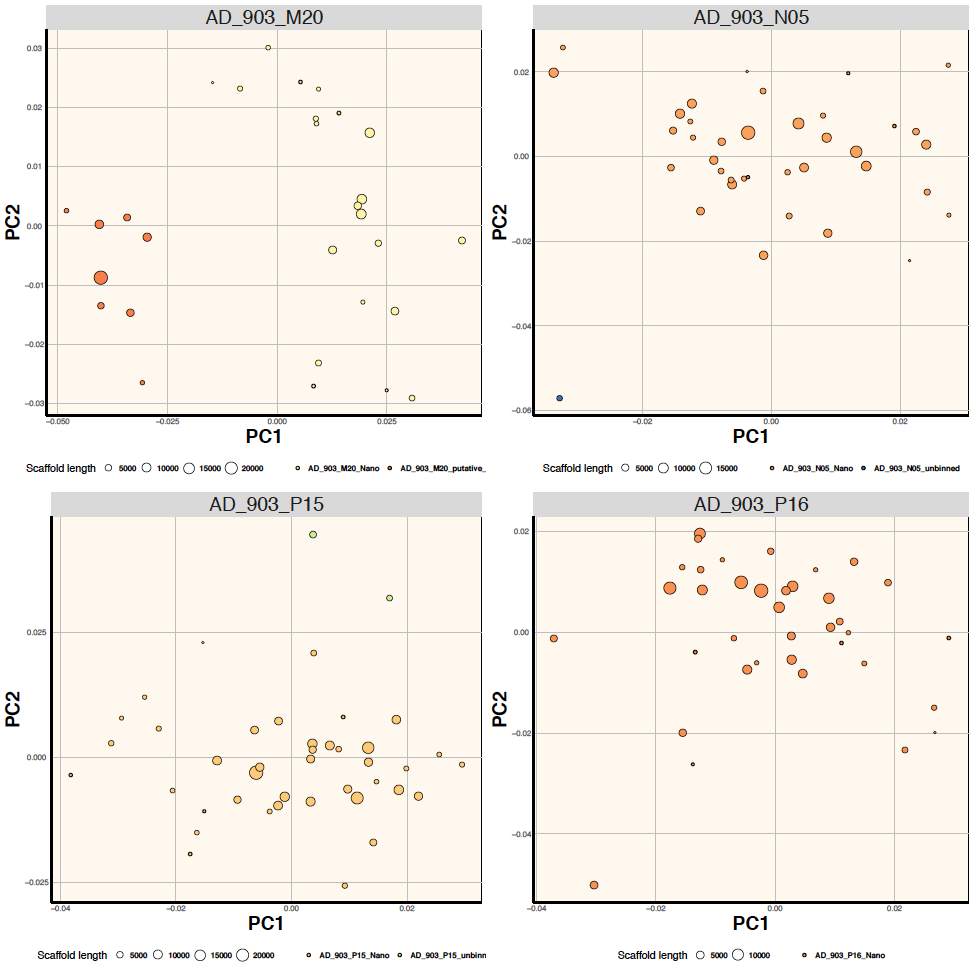
**

**
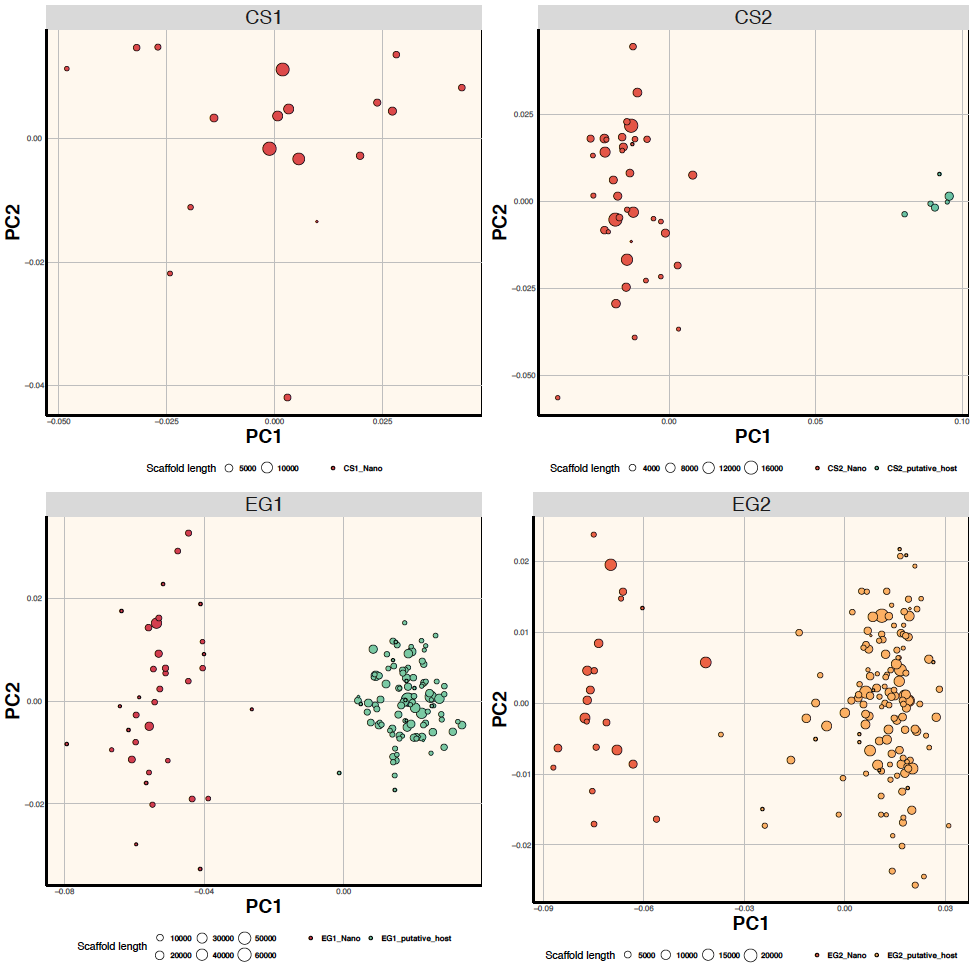
**

**
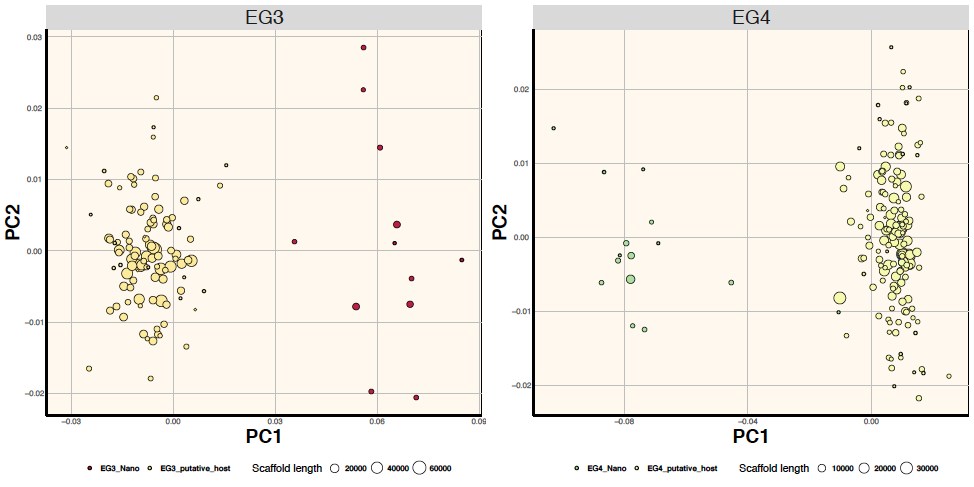
**

**Figure S2. TNF PCA plots for SAGs illustrating separation of *Nanoarchaeota* and putative host genome bins.**

Scaffolds were clustered with PCA based on TNF signatures. Points are scaled by scaffold length and colored by assigned bin. Some SAGs contained only a *Nanoarchaeota* genome bin, but all co-sorted SAGs have well-separated genome bins.

**Figure S3. Maximum likelihood phylogeny of phylum *Nanoarchaeota* based on 16S rRNA gene sequences at least 400 nt in length.**

Sequence names are colored by sampling habitat as shown, red names indicate reference genomes and SAGs included in this study. Node support values from 100 bootstraps shown. *Candidatus* Mancarchaeum acidiphilum is included as an outgroup and is not part of phylum *Nanoarchaeota*.


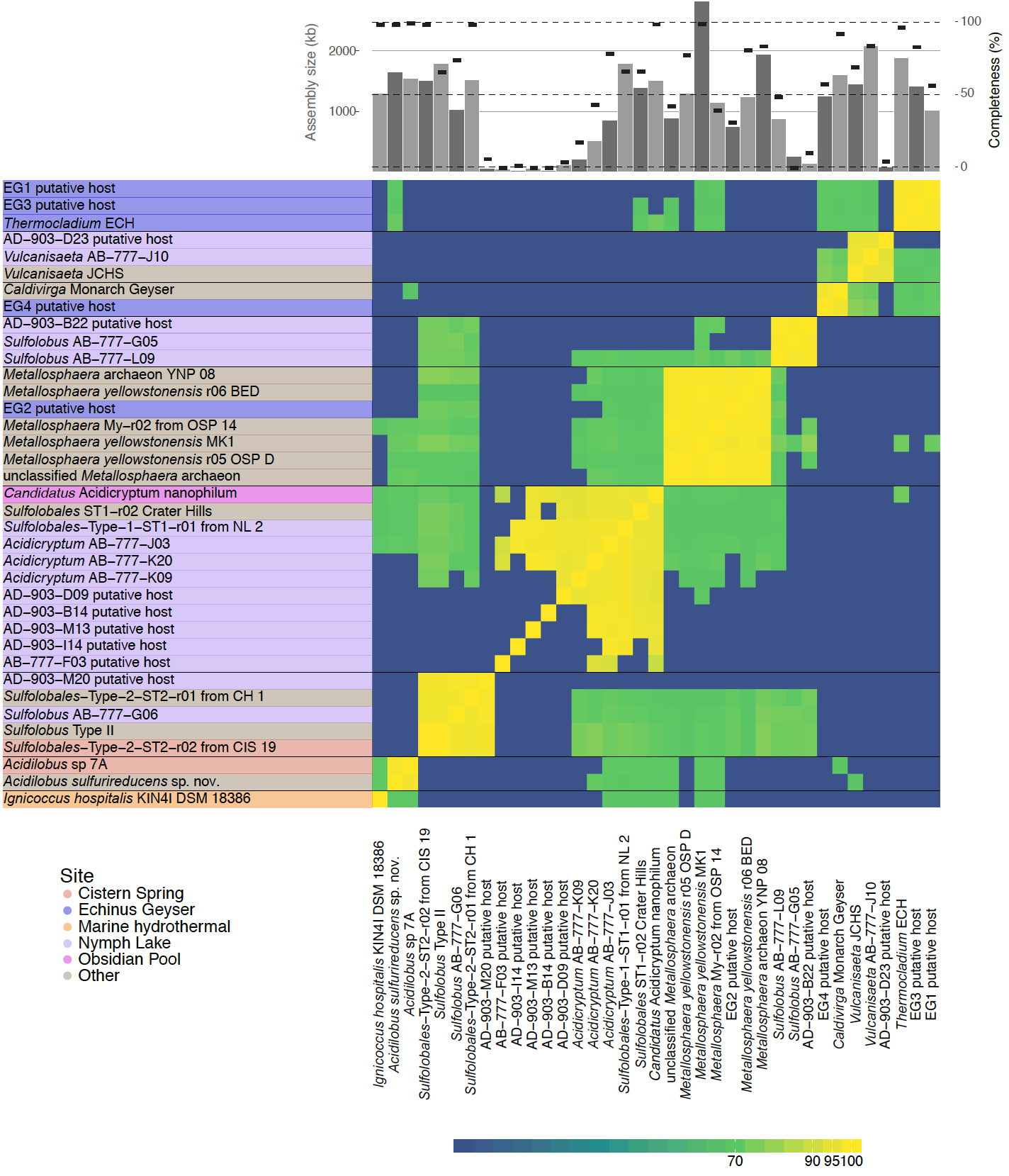


Figure S4. **Identification of putative host genome bins based on ANI to reference genomes and metagenome bins.**

ANI comparisons with alignment lengths less than 20 kb were set to 0% ANI. Black lines between genome bin or genome names delineate groups with at least 95% ANI, and genomes are colored by sampling location. Assembly size (bars) and estimated completeness of genomes (dashes) is shown on top. Complete identifiers for reference genomes and metagenome bins appear in Additional file 2, Table S2. ANI data shown in Additional file 2, Table S8.


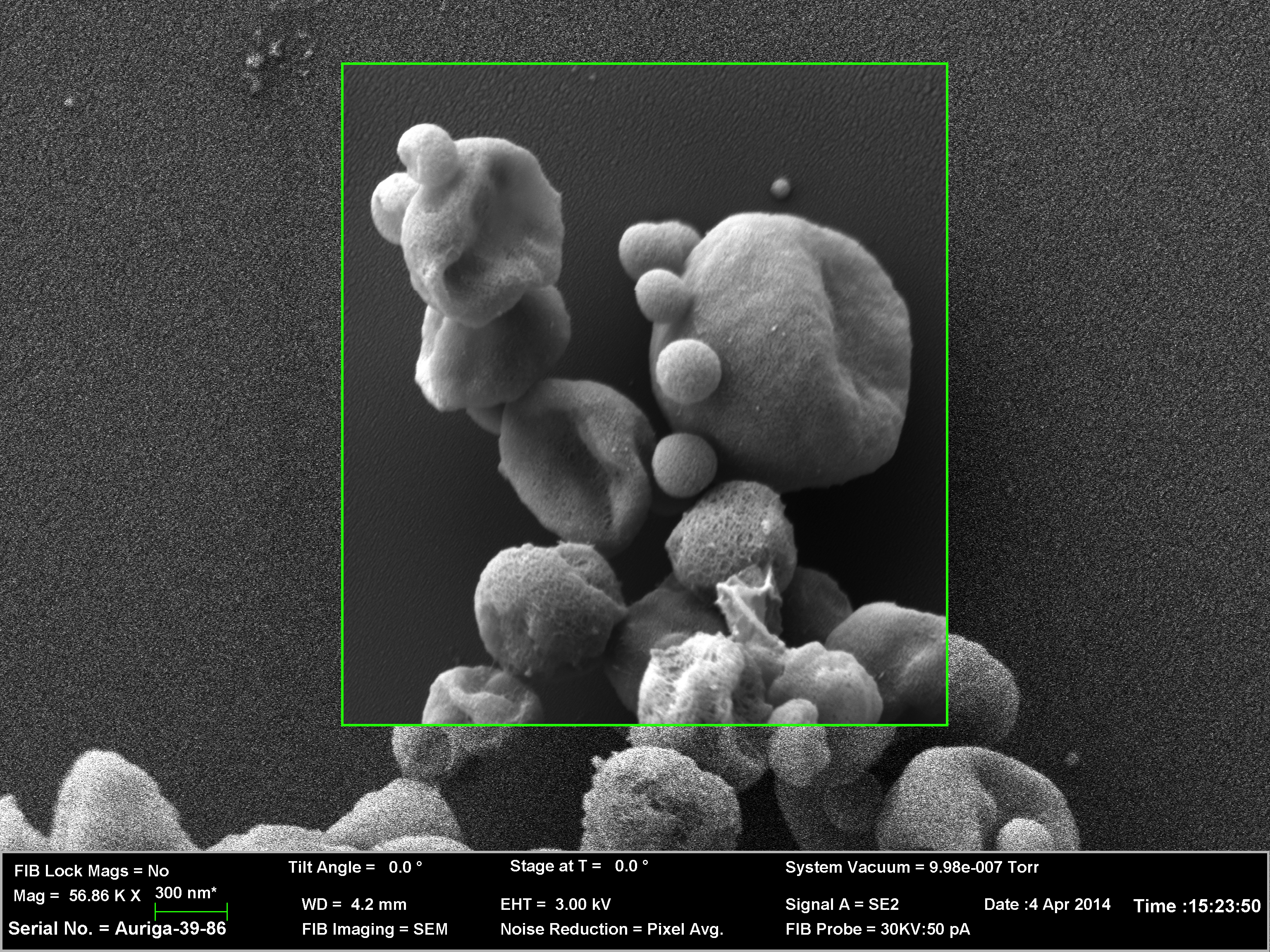


Figure S5. **Scanning electron micrograph of multiple *Nanoarchaeota* cells attached to host cells.** *Nanopusillus acidilobi* on *Acidilobus* sp. 7A is shown. Note linear arrangement of symbionts, consistent with repeated division along a single plane. Isolation, culture conditions, and microscopy procedures as previously described [16].


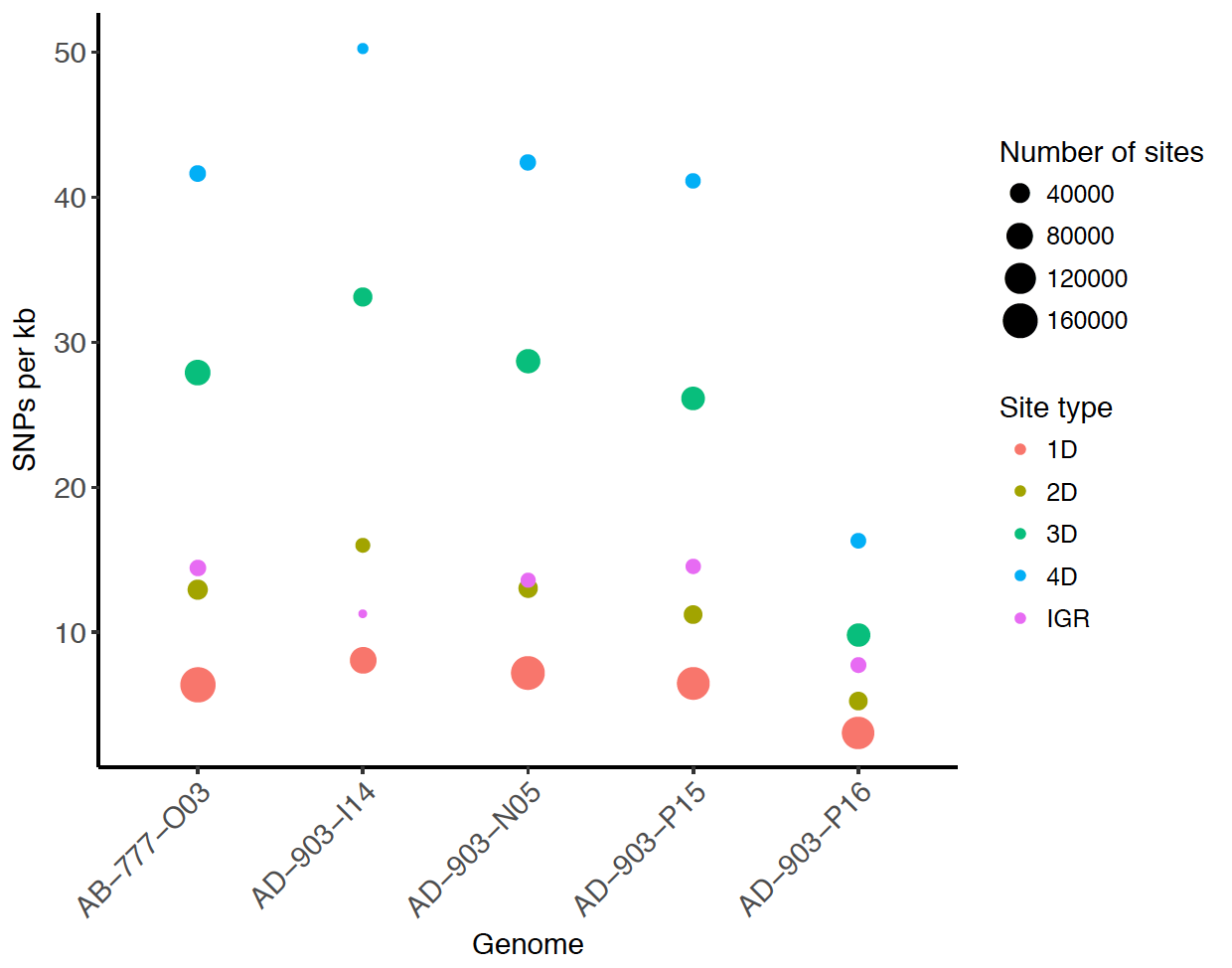


**Figure S6.** **SNP type and density in individual Clade 1 *Nanoarchaeota* SAGs.**

SAG AB-777-F03 was used as a reference to determine the site type (coding or intergenic region (IGR), and fold-degeneracy for coding regions). Point size is scaled to the number of sites in the reference covered by each SAG, and SNPs per kb is calculated based on the number of sites of each type, not the total number of sites. Fold-degeneracy in site type indicates how many possible nucleotides at this position specify the same amino acid. 1D indicates non-synonymous SNPs and 4D synonymous SNPs; whether a SNP at a 2D or 3D site resulted in an amino acid change was not investigated, and these sites were not included in sSNP or nSNP density calculations.


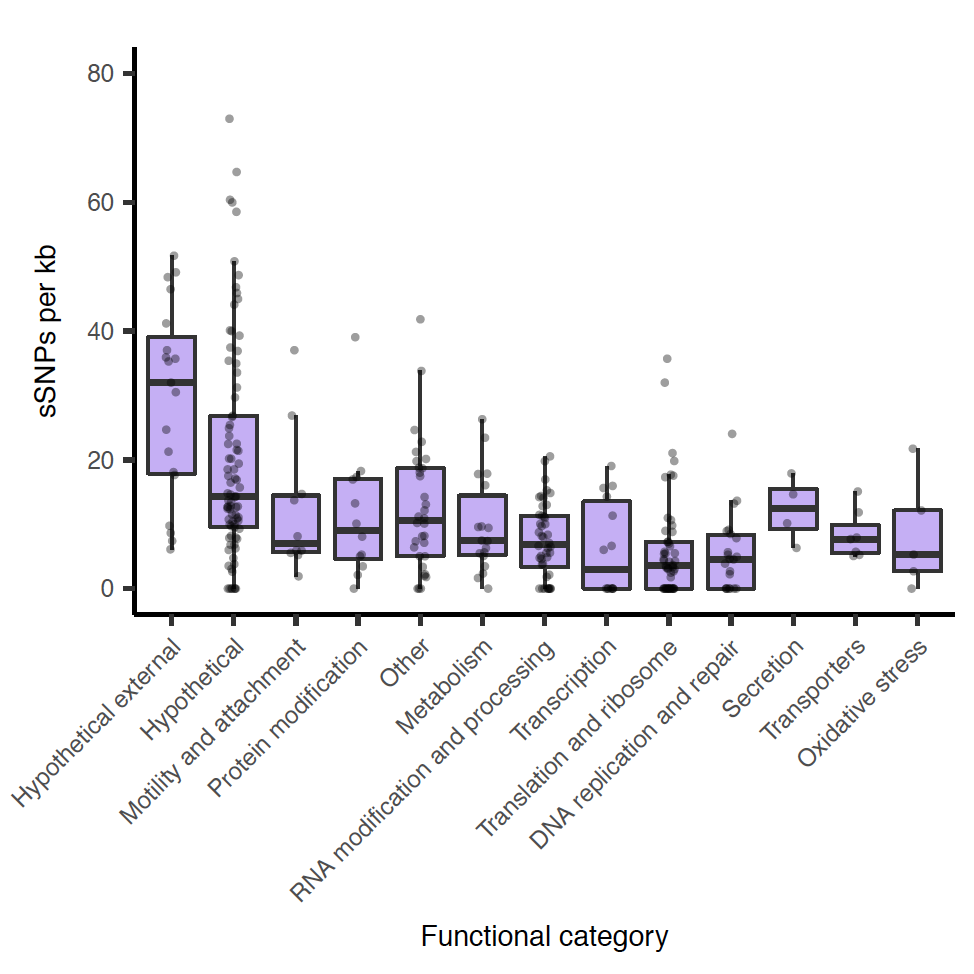


**Figure S7. Variation in sSNP density in Clade 1 *Nanoarchaeota* genes by functional category.**

Box-and-whisker plots of synonymous SNPs per kb of mapped sites per gene, summarized by gene functional category. There were no significant differences in the density of sSNPs between functional categories (one-way ANOVA, F(9, 297) = 0.989, *p* = 0.449). Categories with fewer than 10 genes (Secretion, Transporters, Oxidative stress) were not included in ANOVA.
